# Defining a threshold above which an adult can be considered to frequently use ambulance services: a retrospective cross-sectional study of emergency calls to an ambulance service in England

**DOI:** 10.29045/14784726.2023.3.7.4.35

**Published:** 2023-03-01

**Authors:** Jason Scott, Eduwin Pakpahan, Benjamin Marlow, Nathan Daxner

**Affiliations:** Northumbria University; Northumbria University; South East Coast Ambulance Service NHS Foundation Trust; South East Coast Ambulance Service NHS Foundation Trust

**Keywords:** cross-sectional studies, emergency medical services, health services misuse

## Abstract

**Objective:**

There is no empirical definition of adult frequent use of ambulance services. This study aimed to define a threshold, and utilise this to explore characteristics of people frequently using services.

**Methods:**

This was a retrospective cross-sectional study in a single ambulance service in England. Routinely collected, pseudo-anonymised call- and patient-level data were collected for two months (January and June 2019). Incidents, defined as independent episodes of care, were analysed using a zero-truncated Poisson regression model to determine a suitable frequent-use threshold, with comparisons subsequently made between frequent and non-frequent users.

**Results:**

A total of 101,356 incidents involving 83,994 patients were included in the analysis. Two potentially appropriate thresholds were identified: five incidents per month (A); and six incidents per month (B). Threshold A produced 3137 incidents from 205 patients, with five patients likely false-positive identifications. Threshold B produced 2217 incidents from 95 patients, with no false-positive identifications but 100 false-negatives compared to threshold A. Regardless of threshold, frequent users compared to non-frequent users had relatively reduced service use between 08:00 and 15:00, were younger and were more likely to receive lower-priority responses (all p < 0.001). We identified several chief complaints indicative of increased frequent use, including chest pain, psychiatric/suicide attempt and abdominal pains/problems.

**Conclusions:**

We suggest a threshold of five incidents per month, with recognition that a small number of patients may be incorrectly identified as using ambulance services frequently. The rationale for this choice is discussed. This threshold may be applicable in wider UK settings and could be used for the routine automated identification of people using ambulance services frequently. The identified characteristics can help inform interventions. Future research should examine applicability of this threshold in other UK ambulance services and countries where patterns and determinants of frequent ambulance use may differ.

## Introduction

Frequent use of healthcare services is a well-recognised international phenomenon, with research particularly focusing on emergency department settings (Greenfield et al., 2020; Korczak et al., 2019; Lago et al., 2019; Procter et al., 2021), though emergency departments do not operate in isolation and have far fewer regular contacts with patients than ambulance services (Snooks et al., 2019). The role of ambulance services, which are often referred to internationally as emergency medical services, has been changing considerably over the past decades, with a greater emphasis on delivering care in the community (Leyenaar et al., 2021; Paulin et al., 2020), and many people not being conveyed to hospital (Campagna et al., 2020). This change also reflects the increasing use of ambulance services for problems that have previously been managed in primary care settings (Booker et al., 2015, 2019), and is further reflected across the broader emergency care system where general practitioners are increasingly co-located in emergency departments (Bonciani et al., 2017; Edwards et al., 2020). Ambulance services, as part of a broader care scope, are therefore increasingly identifying people who contact the service frequently (Snooks et al., 2019), which is occurring during a sustained period of increased ambulance service demand that exceeds population growth (Andrew et al., 2020).

Research has identified that frequent use of ambulance services often occurs because people have unmet health or social care needs. For instance, a cross-sectional survey of people calling 911 frequently in a single Canadian city identified that mobility problems, pain and discomfort, anxiety and depression and loneliness were prevalent among the population (Agarwal et al., 2019). Another Canadian study, using qualitative methods, identified that similar complex and often interlinked health (physical and mental) issues and social conditions contributed to frequent use, including limited access to alternative services (Mahmuda et al., 2020). Poor access to other services, particularly outside of regular working hours, has also been hypothesised to be a factor in increased use of emergency medical services in England (Scott et al., 2014a). Other countries where similar issues have been identified include, but are not limited to, Denmark (Søvsø et al., 2019), Netherlands (Maruster et al., 2021) and Singapore (Kuek et al., 2019). Furthermore, mental health, specifically anxiety, and social isolation were identified by emergency medical service providers in the United Kingdom to have been contributing factors in changes to frequent use during the early stages of the COVID-19 pandemic (Scott et al., 2021).

Systematic reviews of frequent ambulance service use identified that there was no single standard definition internationally for adult (Scott et al., 2014b) or paediatric patients (Scott et al., 2023), and a recent commentary on the topic by Brown et al. (2019) called for standardised definitions of frequent use of ambulance services. The aim of this study was to develop a threshold of adult frequent use by evaluating at what point frequent ambulance service use deviated from expected usage, and once the threshold had been identified to compare call characteristics of frequent and non-frequent users.

## Methods

### Design and setting

This study used a retrospective cross-sectional study design based in a single ambulance service in England that uses the advanced medical priority dispatch system (International Academies of Emergency Dispatch, 2021) for triaging emergency calls.

### Data collection

Routinely collected, pseudo-anonymised whole-population call data were collected, with a unique non-identifiable identification number (ID) assigned to each patient. For this anonymisation the ambulance service converted patient National Health Service (NHS) numbers into non-reversible IDs. Where a call did not have an NHS number or where the patient was < 18 years of age, the data were not included. Data included two full months of calls, from January 2019 and June 2019. These months were chosen by the research team as they were considered to allow for seasonal differences in the demand placed on the service and potential differences in the profile of patients while balancing resources required to collect and analyse data. Anonymisation was conducted independently between the two months, ensuring independence of IDs. No linkage was performed between the two months, thus each was treated as independent. Repeat calls relating to the same incident were excluded from the dataset at the point of data collection, therefore an assumption was made that calls and incidents were independent for the purposes of analyses.

Other call-level variables collected included the date and time, response category assigned, chief complaint and despatch code. Response categories were assigned one of two codes: a ‘category’ code where there was no healthcare professional (HCP) involved in making the call, and an ‘HCP admission protocol’ where a HCP was involved. Within each of these two codes there were four levels of severity (1–4), with 1 being most urgent and 4 being least urgent. These four levels are defined as:

1. an immediate response to a life-threatening condition;
2. a serious condition which may require rapid assessment and/or urgent transport;
3. an urgent problem which requires treatment and transport to an acute setting; and
4. a non-urgent problem which requires transportation to a hospital ward or clinic.

Patient-level variables included age, sex, total number of calls received and total number of incidents. A call only became an incident once it reached classification for being ‘hear and treat’, ‘see and treat’ or ‘see and convey’; therefore, repeat calls for the same reason (such as checking on progress of an ambulance) would not be classed as a new incident. An incident is therefore defined as a single independent episode of care. All calls without incidents were excluded from the study. The patient’s age was assigned at the point of the first call only.

### Data analysis

Data were analysed following the approach developed by Locker et al. (2007) for determining a definition of frequent use of emergency departments. We analysed data on a monthly basis, as the purpose was to update the monthly definition of frequent use that is operationalised by ambulance services in the United Kingdom. In our analysis there were no zero counts, and therefore the proper analysis we consider is the zero-truncated Poisson (ZTP) regression. This model is appropriate when the data are from a mechanism (calls to an ambulance service) in which zero counts do not exist in the dataset. The probability of zero count, exp (-λ), is taken into account by the probability of not having the zero count, which is 1-exp (-λ). Dividing that Poisson distribution for the positive outcomes results in the ZTP. The ZTP distribution is:


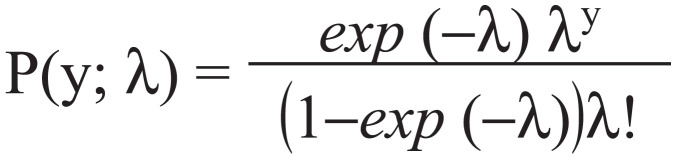


where y = 1,2, . . . and λ > 0. This distribution only allows the positive integer, and in our case was those who made at least one call.

#### Stage 1

The first stage of analysis was to identify whether all incidents are independent, which was determined by analysing the mean rate of incidents, then multiplying the probability by the total number of patients within the study. This was then plotted on a logarithmic scale and compared against the expected (ZTP) distribution. Predictive margin plots were also created using age and sex variables. The ZTP model was then used to identify a suitable threshold for frequent use, where the number of expected patients was negligible.

#### Stage 2

Once the threshold for frequent use had been identified, the second stage of analysis consisted of categorising incidents as being from patients who were either below (non-frequent callers) or above (frequent callers) this threshold. Comparisons were then made between incidents from people calling frequently and people not calling frequently based on call-level variables of age, sex, category code and chief complaint. As the first stage resulted in uncertainty in the threshold, this comparison included two potentially suitable thresholds, defined as:

- Threshold A: five or more incidents per month
- Threshold B: six or more incidents per month

The rationale for these two thresholds is presented in the results based on stage 1 of the analysis, and clinical implications are deliberated in the discussion. The comparisons included time-series analyses by time of day (time data converted into hourly groups), independent samples t-test (age) and Chi-squared (sex, category code, chief complaint) analyses. We conducted the inferential analyses based on the assumption that the within number of calls are independent. A descriptive comparison of the difference in proportional number of incidents based on category code and chief complaint was also produced. The alpha level was set at 0.05 for all inferential analyses.

## Results

**Figure 1:**
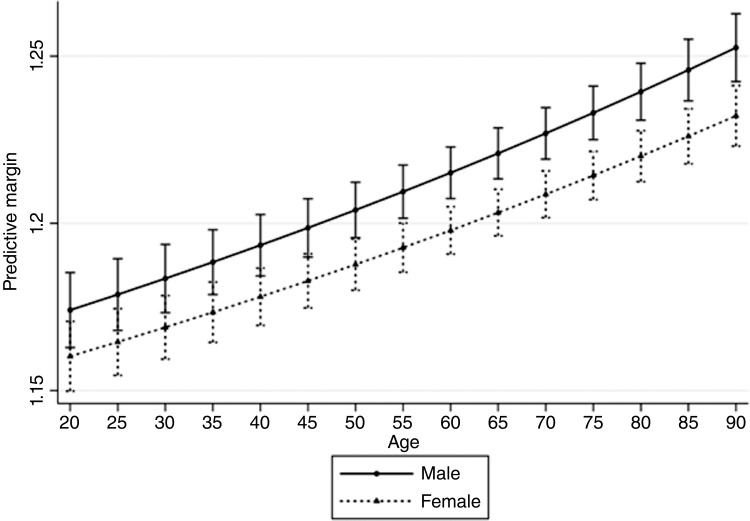
Figure 1. Predictive margin plot for number of incidents based on age and sex.

A total of 83,994 patients were included in the study, and they were involved in a total of 101,356 incidents in January 2019 (n = 52,813) and June 2019 (n = 48,543). The mean age of patients was 63.5 years (range 18 to 104, standard deviation (SD) = 22.7) and more patients were female than male (55.0%; valid n = 46,161). A predictive margin plot for number of incidents (Figure 1) shows that older adults are significantly more likely to have more incidents. Prior to the age of 50, there is no significant difference between males and females, but after age 50 males are involved in significantly more incidents than females.

### Frequent and non-frequent incidents

**Figure 2:**
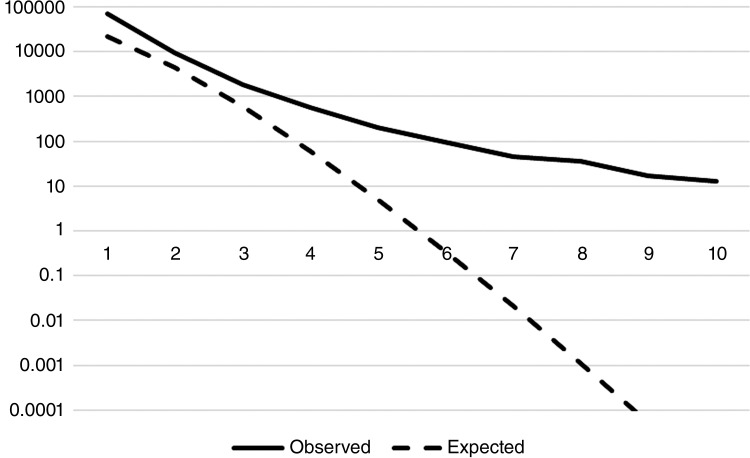
Figure 2. Truncated Poisson distribution showing observed and expected frequency of patients (up to 10 incidents/month).

Table 1 and Figure 2 present the observed and predicted frequencies of incidents. Within the dataset, threshold A resulted in 3137 (3.1%) incidents related to frequent use, compared to 2217 (2.2%) incidents using threshold B. Threshold A results in 205 patients being identified as frequent callers, of which 200 (97.6%) would be true-positive cases, and five (2.4%) would be expected to call, thus representing false-positive cases. Threshold B results in 95 patients (100%) being identified as true-positive cases, with zero false-positive cases (expected frequency = 0.35). However, using threshold B would result in 105 false-negative cases compared with threshold A. There is therefore some uncertainty as to whether threshold A or B would be the most appropriate. We have included results of stage 2 analyses for both thresholds and return to the implications in the discussion.

**Table 1.** Observed and expected frequency of patients per number of incidents (up to 10 incidents/month).

Number of incidents	Observed patients	Expected patients
1	71,805	22,278.753347
2	9293	4445.206063
3	1858	598.944415
4	567	61.248425
5	205	5.064838
6	95	0.352775
7	45	0.021250
8	35	0.001126
9	17	0.000054
10	13	0.000002

### Characteristics of people using the service frequently: time series

**Figure 3:**
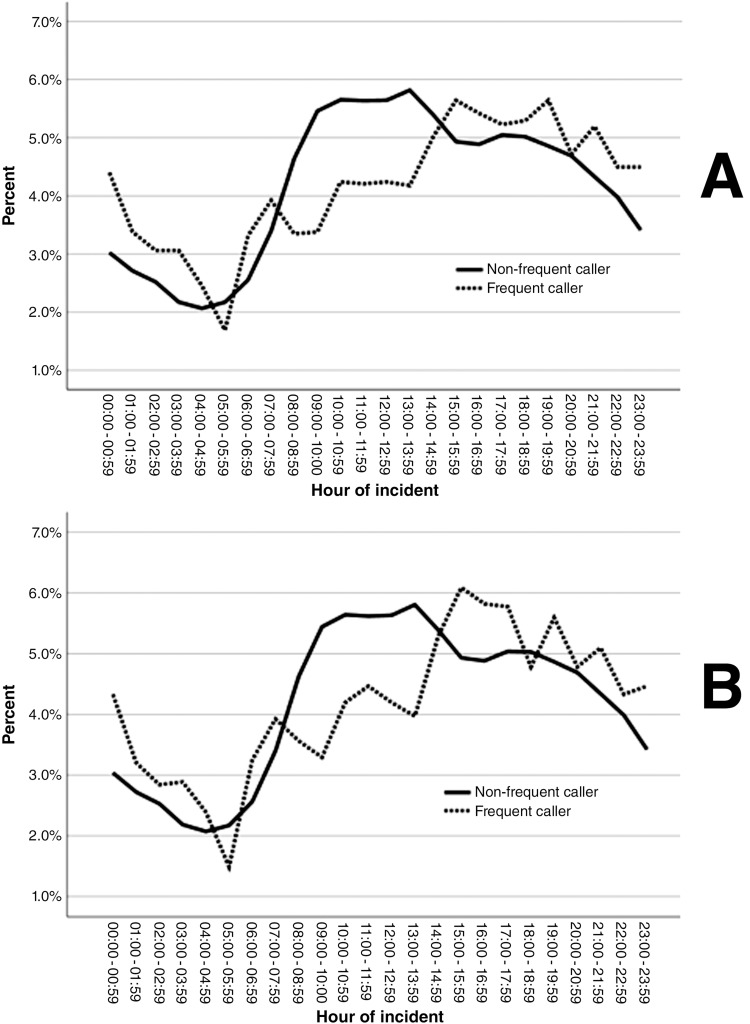
Figure 3. Time-series data based on a threshold of five or more incidents per month (A) and six or more incidents per month (B).

There were observable differences in the time-series data based on hour of incident. Figure 3 presents the differences based on a threshold of five incidents per month (A) and six incidents per month (B), of which there is little difference between them. Notably, across both thresholds, incidents among those calling frequently compared to those not calling frequently were consistently lower between the hours of 8:00 and 15:00, with another single deviation at 5:00.

### Call-level characteristics of people using the service frequently

Comparisons between frequent users and non-frequent users using call-level variables (age, sex, category code and chief complaint) across the two thresholds are presented in Table 2.

**Table 2.** Comparison of call-level characteristics of people calling frequently with those not calling frequently.

Variable	Threshold						Difference in proportion of incidents
	A: Five or more incidents per month			B: Six or more incidents per month			
	Non-frequent callers	Frequent callers	Significance^a^ or % difference	Non-frequent callers	Frequent callers	Significance^a^ or % difference	
**Mean age (SD)**	64.1 (22.5)	59.4 (21.9)	<** 0.001**	64.1 (22.5)	57.1 (21.0)	<** 0.001**	
**Female (%)**	53,650 (54.7)	1729 (55.1)	**0.626**	54,116 (54.6)	1263 (57.0)	**0.029**	
**Category code**			<** 0.001**			<** 0.001**	
*Category 1*	6233 (6.3%)	179 (5.7%)	−0.6%	6276 (6.3%)	136 (6.1%)	−0.2%	+0.4%
*Category 2*	56,890 (57.9%)	1561 (49.8%)	−8.1%	57,349 (57.8%)	1102 (49.7%)	−8.1%	0.0%
*Category 3*	24,122 (24.6%)	941 (30.0%)	+5.4%	24,396 (24.6%)	667 (30.1%)	+5.5%	+0.1%
*Category 4*	5038 (5.1%)	460 (11.5%)	+6.4%	5135 (5.2%)	263 (11.9%)	+6.7%	+0.3%
*HCP admission protocol 1*	709 (0.7%)	18 (0.6%)	−0.1%	717 (0.7%)	10 (0.5%)	−0.3%	−0.2%
*HCP admission protocol 2*	741 (0.8%)	12 (0.4%)	−0.4%	750 (0.8%)	3 (0.1%)	−0.7%	−0.3%
*HCP admission protocol 3 or 4^b^*	4486 (4.6%)	66 (2.1%)	−2.5%	4516 (4.6%)	36 (1.6%)	−2.9%	−0.4%
**Chief complaint^c^**			<** 0.001**			<** 0.001**	
*Abdominal pains/problems*	1910 (1.9%)	105 (3.3%)	+1.4%	1928 (1.9%)	87 (3.9%)	+2.0%	+0.6%
*Back pains (non-traumatic)*	690 (0.7%)	35 (1.1%)	+0.4%	704 (0.7%)	21 (0.9%)	+0.2%	−0.2%
*Breathing problems*	8586 (8.7%)	243 (7.7%)	−1.0%	8670 (8.7%)	159 (7.2%)	−1.5%	−0.5%
*Chest pain*	7985 (8.1%)	402 (12.8%)	+4.7%	8049 (8.1%)	338 (15.2%)	+7.1%	+2.4%
*Convulsions/fitting*	2113 (2.2%)	113 (3.6%)	+1.4%	2126 (2.1%)	100 (4.5%)	+2.4%	+1.0%
*Diabetic problems*	909 (0.9%)	40 (1.3%)	+0.4%	917 (0.9%)	32 (1.4%)	+0.5%	+0.1%
*Escalate / fail to escalate*	8825 (9.0%)	312 (9.9%)	+0.9%	8942 (9.0%)	195 (8.8%)	−0.2%	−1.1%
*Falls*	5753 (5.9%)	152 (4.8%)	−1.1%	5831 (5.9%)	74 (3.3%)	−2.6%	−1.5%
*HCP admission/transfer*	8500 (8.7%)	114 (3.6%)	−5.1%	8555 (8.6%)	59 (2.7%)	−4.9%	+0.2%
*Haemorrhage/lacerations*	3097 (3.2%)	126 (4.0%)	+0.8%	3138 (3.2%)	85 (3.8%)	+0.6%	−0.2%
*Heart problems / AICD*	1185 (1.2%)	41 (1.3%)	+0.1%	1187 (1.2%)	39 (1.8%)	+0.6%	+0.5%
*NHS Pathways*	25,485 (25.9%)	554 (17.7%)	−8.2%	25,688 (25.9%)	351 (15.8%)	−10.1%	−1.9%
*Overdose/poisoning (ingestion)*	1741 (1.8%)	81 (2.6%)	+0.8%	1775 (1.8%)	47 (2.1%)	+0.3%	−0.5%
*Psychiatric / suicide attempt*	1726 (1.8%)	262 (8.4%)	+6.6%	1758 (1.8%)	230 (10.4%)	+8.6%	+2.00%
*Sick person*	5374 (5.5%)	224 (7.1%)	+1.6%	5440 (5.5%)	158 (7.1%)	+1.6%	0.00%
*Stroke*	2467 (2.5%)	49 (1.6%)	−0.9%	2482 (2.5%)	34 (1.5%)	−1.0%	−0.1%
*Traumatic injuries, specific*	3865 (3.9%)	82 (2.6%)	−1.3%	3889 (3.9%)	58 (2.6%)	−1.3%	0.0%
*Unconscious/fainting*	3817 (3.9%)	60 (1.9%)	−2.0%	3837 (3.9%)	40 (1.8%)	−2.1%	−0.1%

^a^Compared with non-frequent callers.

^b^Only three incidents by non-frequent callers were assigned HCP admission protocol 3, so the data were combined with HCP admission protocol 4.

^c^Only chief complaints constituting ≥ 1% of incidents within any single category are included in the table. All chief complaints were included in the statistical analyses.

AICD: automated implantable cardioverter defibrillator; HCP: healthcare professional; NHS: National Health Service; SD: standard deviation.

#### Age

An independent samples t-test comparing age of non-frequent callers (mean = 64.1, SD = 22.5) with frequent callers defined as five or more incidents per month (threshold A; mean = 59.4, SD = 21.9) identified that people calling frequently were significantly younger by 4.7 years (95% confidence interval (CI) = 3.9 to 5.5) than people not calling frequently (t = 11.841, df = 3352.4, p < 0.001). This difference was larger using threshold B, where people calling frequently (mean = 57.1, SD = 21.0) were significantly younger than people not calling frequently (mean = 64.1, SD = 22.5) by 7.0 years (95% CI = 6.1 to 7.9; t = 15.486, df = 2331.4, p < 0.001).

#### Sex

There were more incidents relating to females than males, whether using threshold A (n = 1729 female, 55.1%; n = 1408 male, 44.9%) or threshold B (n = 1263 female, 57.0%; n = 954 male, 43.0%). Comparing proportions of incidents involving females between frequent and non-frequent use, using threshold A, people who called frequently were not significantly more likely to be female (n = 1729, 54.7%) than those who did not call frequently (n = 53,650, 54.8%; x2 = 0.238, df = 1, p = 0.626). Using threshold B, people who called frequently were significantly more likely to be female (n = 1263, 57.0%) than those who did not call frequently (n = 51,116, 54.6%; x2 = 4.753, df = 1, p = 0.029).

#### Category code

When using threshold A, there was a significant difference (x2 = 350.993, df = 6, p < 0.001) in the category code assigned to callers. A lower relative proportion of people calling frequently received a category 2 or HCP admission protocol 4 code, and a higher proportion received a category 3 or category 4 code. This same pattern presented when using threshold B (x2 = 288.226, df = 6, p < 0.001). The difference in category 1 calls across both thresholds between frequent and non-frequent users was relatively small. Comparing differences in proportional incidents between thresholds A and B identified no change of > 1% in category codes assigned to calls.

#### Chief complaint

There were significant differences in chief complaint when using both threshold A (x2 = 1201.684, df = 39, p < 0.001) and threshold B (x2 = 1432.083, df = 39, p < 0.001), with similar patterns observed across almost all chief complaints. The largest relative increases in proportion of calls among frequent caller groups (thresholds A and B) were for chest pain (4.7% and 7.1%, respectively), psychiatric / suicide attempt (6.6% and 8.6%), convulsions/fitting (1.4% and 2.4%), abdominal pain/problems (1.4% and 2.0%), and sick person (1.6% and 1.6%). The largest decreases in proportion of calls among frequent caller groups (thresholds A and B) were observed for NHS Pathways (8.2% and 10.1%), HCP admission/transfer (5.1% and 4.9%), falls (1.1% and 2.6%) and unconscious/fainting (2.0% and 2.1%).

## Discussion

This was the first study internationally to develop a threshold of adult frequent use of ambulance services, addressing a well-recognised gap in the literature (Brown et al., 2019; Scott et al., 2014b). We identified two potential thresholds for frequent use – five incidents per month or six incidents per month – where an incident is defined as a single episode of care that receives a ‘hear and treat’, ‘see and treat’ or ‘see and convey’ response. We propose that the most suitable threshold is five incidents per month. This threshold in an average month, based on the single ambulance service in which we collected data, would result in 205 patients being identified as using the service frequently, of which five (2.4%) would be false-positive identifications, though the generalisability of these findings is unknown. To address this, services should continue to conduct patient reviews prior to any automated intervention.

A single threshold is useful for developing and operationalising automated systems for identifying people who are frequently using services. However, a threshold would not fully take into account the complexity of reasons for frequent service use. Other case-finding approaches could supplement automated systems, such as identifying patients with complex needs (Hudon et al., 2021). Automated identification systems may therefore introduce missed opportunities to intervene and deliver the various interventions that are currently in use for people who use ambulance services frequently (Snooks et al., 2019). Such interventions have been associated with reductions in service utilisation (Edwards et al., 2015), but there is a need to determine their cost effectiveness. Research is currently ongoing to examine the cost effectiveness of case management (Aslam et al., 2022), and further work will be required to refine approaches to case-finding which incorporate a better understanding of the wider determinants of health and how they contribute to frequent use.

Using the threshold of five incidents per month, we identified that frequent use changes depending on time of day, specifically with a large relative decrease in calls by people who call frequently between the hours of 08:00 and 15:00, compared to patients who do not call frequently. This finding contributes to the increasing literature that identifies limited access to other services as a contributing factor in frequent ambulance service use (Agarwal et al., 2019; Mahmuda et al., 2020), and includes very similar patterns seen in previous research (Scott et al., 2014a). People who frequently use ambulance services are often users of multiple regional services, which has been described as a potential inefficient use of resources (Maruster et al., 2020). There is a need for future research to examine why a lack of access to other services increases frequent ambulance service use, and this would be a potential avenue for future intervention development. We also identified that a larger proportion of frequent users received a lower acuity response than non-frequent users. Again, this supports findings from previous research on frequent ambulance service use (Scott et al., 2014a), but could also be explained by ongoing management of people calling frequently being a confounding factor; patients with a management plan may have had their level of response adjusted following clinical assessment within the study’s sample.

We identified several chief complaints indicative of increased frequent use, including chest pain, psychiatric / suicide attempt and abdominal pains/problems. It should be noted that the data were based on call disposition only, and the accuracy of these dispositions is unknown. The evidence base suggests that poor mental health is a significant contributing factor in frequent use of healthcare services (Soril et al., 2016), and therefore the 8.4% of incidents identified as being psychiatric / suicide attempt, which is the only disposition related solely to mental health, is likely to be a large underestimate of the extent that poor mental health is contributing to frequent use, or the complex interaction that exists between the triad of physical health, mental health and social conditions (Kuek et al., 2019; Scott et al., 2021; Søvsø et al., 2019; Urbanoski et al., 2018). Similarly, none of the dispositions are able to identify social conditions or wider determinants of health which are known to contribute to frequent use (Agarwal et al., 2019; Kuek et al., 2019; Mahmuda et al., 2020).

Finally, the threshold developed in this study was based on adult (≥ 18 years) frequent use of an ambulance service. There has been very little published on paediatric frequent use of emergency medical services (Scott et al., 2023), and this requires further attention from both practice and research perspectives.

### Limitations

An important limitation of our study was that the developed Poisson regression model incorporated age and sex data only. Ambulance services in England obtain little routinely collected patient-level data that could be incorporated in the model, and the study team did not have the resources to collect additional data via data linkage, nor did we have access to primary clinical impression. Furthermore, a large number of incidents were coded as ‘NHS Pathways’, which is a referral from NHS111. NHS111 provides telephone-based urgent care services and uses a separate triage system, meaning that the coding of these calls is not indicative of the patient’s underlying medical condition, other than that the patient deemed it to be urgent rather than an emergency. Future research exploring definitions of frequent ambulance service or EMS use should incorporate more robust data on patient outcomes beyond disposition codes, which have relatively limited sensitivity depending on the medical condition (Green et al., 2019; McClelland & Burrow, 2021). This should also include a comparison between disposition codes and on-scene clinical impressions.

Another limitation was that data were obtained from a single ambulance service in England, thus the generalisability of the results to other ambulance services in the United Kingdom or other countries is unknown and further demonstrates the need for replication. There are numerous models of emergency medical systems internationally (Al-Shaqsi, 2010), and the evidence base is unclear whether funding models or other determinants influence service demand (Ting & Chang, 2006; Tippett et al., 2012). It is therefore possible that different models may influence ambulance demand, which in turn may influence thresholds for frequent use. This requires further study, ideally using comparative data across multiple emergency medical systems in multiple countries. Finally, data were only collected for two separate months due to limited study resources. Future research should consider including data over a whole year period, which would allow for an understanding of how usage patterns change over time.

## Conclusion

We suggest a threshold of five incidents per month could be used in a UK setting to identify people who use ambulance services frequently, with recognition that a small number of patients may be incorrectly identified. Future research should examine applicability of this threshold in other ambulance services and in other countries where patterns and determinants of frequent ambulance use may differ.

## Author contributions

JS conceived and designed the study. JS, BM and ND collected the data. JS and EP analysed the data, with JS, BM, EP and ND interpreting the results. JS drafted the manuscript and all authors contributed substantially to its revision. JS acts as the guarantor for this article.

## Conflict of interest

None declared.

## Ethics

The study received ethical approval from Northumbria University’s Ethics Online system (ref: 28990).

## Funding

None.
